# Efficacy of perioperative repetitive transcranial magnetic stimulation for postoperative depression in breast cancer patients: study protocol for a single-center randomized controlled trial

**DOI:** 10.3389/fpsyt.2026.1923066

**Published:** 2026-09-14

**Authors:** Sitong Zhou, Wenhui Wang, Shirong Wei, Junwen Tu, Tong Zhi, Ming Yao, Huadong Ni

**Affiliations:** 1 Department of Anesthesia Medicine, Zhejiang Chinese Medical University, Hangzhou, Zhejiang, China; 2 Department of Anesthesiology and Pain Research Center, The Affiliated Hospital of Jiaxing University, Jiaxing, China

**Keywords:** breast cancer, HAMD-17, postoperative depression, randomized controlled trial, rTMS

## Abstract

**Background:**

Breast cancer (BC) is one of the most prevalent malignancies worldwide, and the perioperative period represents a vulnerable phase during which surgical stress and psychological burden may contribute to postoperative depression and impaired recovery. Repetitive transcranial magnetic stimulation (rTMS) is an established non-invasive neuromodulation technique for depressive disorders; however, evidence regarding its application for preventing postoperative depression (PD) in patients with breast cancer remains limited.

**Methods/design:**

This single-center, prospective, randomized controlled study will enroll 182 patients scheduled for breast cancer surgery with preoperative depressive symptoms defined as a 17-item Hamilton Depression Rating Scale (HAMD-17) score ≥7. Participants will be randomly allocated to receive either active rTMS or sham stimulation. Active rTMS will be delivered over the left dorsolateral prefrontal cortex (DLPFC) at 10 Hz and 120% of the resting motor threshold. Two sessions of rTMS will be administered during the perioperative period, including one session one day before surgery and one session before surgery on the day of operation. Sham stimulation will mimic acoustic and sensory characteristics of active stimulation without delivering an effective magnetic field to the cortex. The primary outcome is the incidence of postoperative depression (PD) at postoperative day 3, operationally defined as the proportion of participants with preoperative depressive symptoms (HAMD-17 ≥7) who have a postoperative HAMD-17 score of 7 or above. Secondary outcomes include longitudinal changes in HAMD-17 scores at postoperative day 1, day 3, day 7, and 3 months, frailty status, sleep quality, postoperative pain intensity, and serum brain-derived neurotrophic factor (BDNF) levels in a randomly selected subset of participants.

**Discussion:**

This trial will evaluate whether perioperative rTMS can reduce the incidence of postoperative depression and improve depressive symptom severity in breast cancer patients undergoing surgery. The findings may provide preliminary evidence regarding the potential efficacy and safety of ultra-brief perioperative rTMS and inform future neuromodulation strategies for perioperative affective symptom management in oncology populations.

**Clinical trial registration:**

## 1 Introduction

Breast cancer (BC) is a major global health challenge and is the most commonly diagnosed malignancy worldwide (1). The significant disease burden is accompanied by a high prevalence of psychological morbidity, particularly in the perioperative period following surgical treatment (2, 3). Postoperative depression and depressive symptoms are commonly observed in patients with breast cancer undergoing surgery (4). These symptoms, characterized by persistent low mood, somatic symptoms, and in severe cases, suicidal ideation, may impair postoperative recovery, reducing quality of life (QoL), and negatively influence long-term functional outcomes (5). Therefore, preventing and managing postoperative depression represents an important component of comprehensive breast cancer care, underscoring the need for effective and well-tolerated interventions.

The development of depressive symptoms during the perioperative period is influenced by complex interactions between biological and psychological processes (6). Neuroinflammation processes has been implicated as one of the potential biological mechanisms involved in depression (7). Surgical trauma and anesthesia can induce systemic inflammatory responses characterized by increased production of pro-inflammatory mediators, including IL-6 and TNF-α (8). These cytokines may influence blood-brain barrier integrity and neural signaling, which have been implicated in affective symptom regulation (9). Concurrently, depressive symptom severity has been associated with reduced brain-derived neurotrophic factor (BDNF) levels (10), a protein essential for neuronal survival, plasticity, and synaptic adaptation (11). This interaction between inflammatory activity and neurotrophic signaling has been proposed to contribute to postoperative depressive symptom development and severity changes. Although pharmacological interventions are available, their clinical application may be limited by factors including adverse effects and variable treatment responses (12), highlighting the need for alternative or adjunctive strategies.

Repetitive transcranial magnetic stimulation (rTMS) is a non-invasive neuromodulation technique that can modulate cortical excitability and functional connectivity within mood-related networks (13, 14). By applying focused magnetic pulses to brain regions involved in emotional regulation (15), rTMS may influence neural circuit dynamics associated with affective symptom regulation (16). Its therapeutic effects have been associated with modulation of neuroplasticity-related pathways, including BDNF signaling (17), although its role in perioperative settings remains insufficiently studied. Its non-invasive nature and favorable safety profile make it a promising candidate for patients undergoing breast cancer surgery (18).

Despite this potential, there is a paucity of rigorous clinical trials evaluating rTMS for postoperative depression in patients with breast cancer undergoing surgery. Most existing studies focus on major depressive disorder in general or treatment-resistant populations (19), rather than postoperative depression in oncology settings. This study aims to address this gap. We hypothesize that perioperative rTMS may reduce the incidence of postoperative depression compared with sham stimulation in patients with breast cancer undergoing surgery. In addition, rTMS may improve longitudinal depressive symptom severity assessed by continuous HAMD-17 scores. The primary objective of this trial is to evaluate whether perioperative rTMS can reduce the proportion of participants meeting the predefined threshold for postoperative depression (PD) at postoperative day 3, as specified in the registered protocol, while secondary objectives include safety assessment and exploratory evaluation of neurobiological markers such as BDNF.

## 2 Methods and analysis

### 2.1 Study design

This study is a prospective, single-center, randomized, sham-controlled clinical trial conducted at the Affiliated Hospital of Jiaxing University. The protocol has been approved by the institutional ethics committee (2025-KY-048) and registered at the China Clinical Trial Registration Center (ChiCTR2500097808). This protocol is reported in accordance with the SPIRIT 2013 guidelines, and the completed SPIRIT 2013 checklist is provided as **Supplementary Material**. **Figure 1** is a flow diagram depicting the process of engaging patients, implementing the intervention, and reviewing the results.

**Figure 1:**
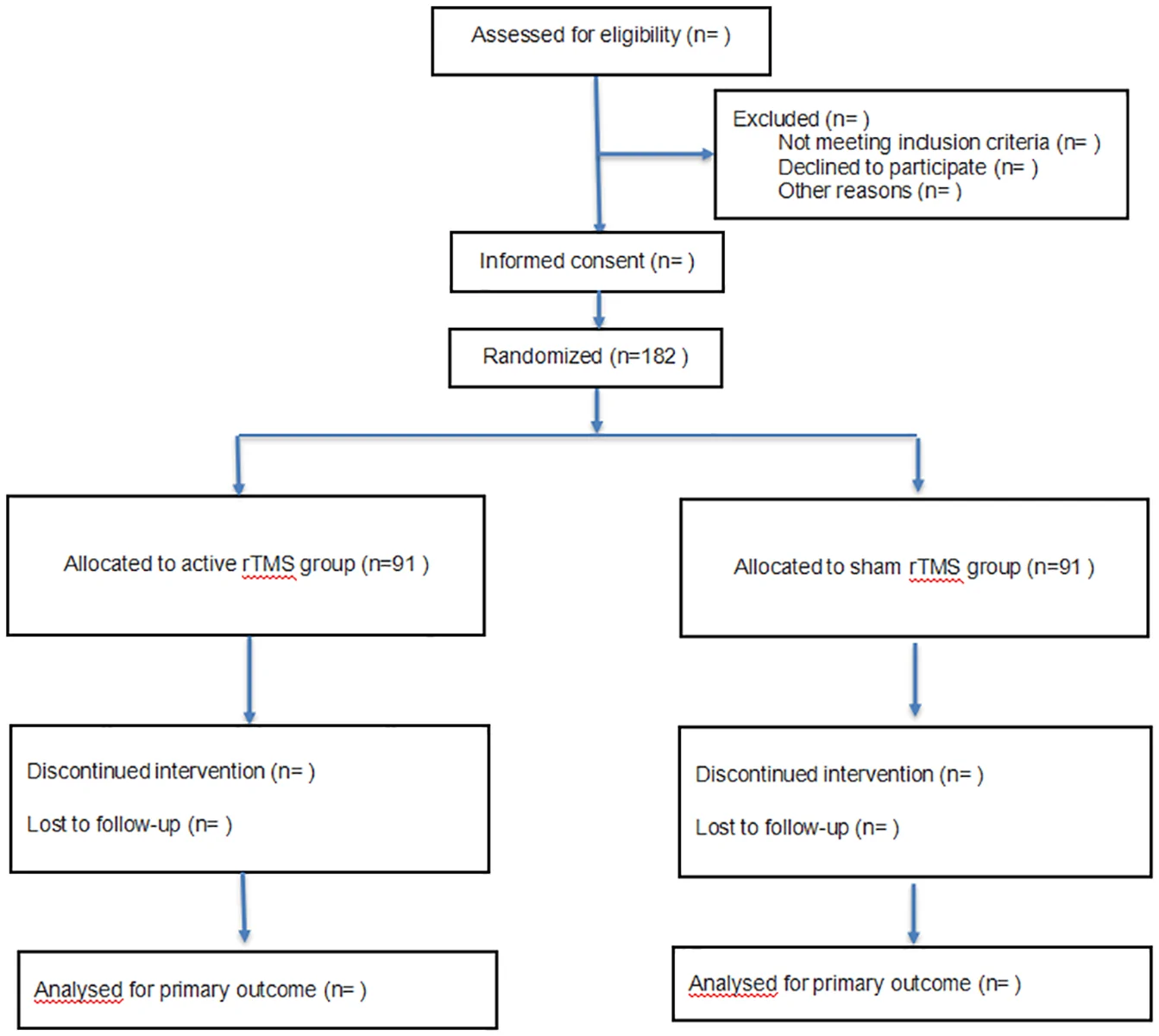
SPIRIT flow diagram of the study design. As this is a protocol study, actual participant numbers for screening, exclusion, and follow-up are not available before trial completion. The numbers presented in the randomized groups represent the planned sample sizes according to the prespecified sample size calculation.

### 2.2 Participants and eligibility criteria

Eligible participants are adults (≥18 years) diagnosed with breast cancer and scheduled for surgical treatment under general anesthesia. All participants must present with preoperative depressive symptoms, defined as a HAMD-17 score ≥7, and must have an ASA physical status classification of I–III. Written informed consent will be obtained prior to enrollment.

### 2.3 Inclusion criteria

Eligible participants are adults aged 18 years or older with an American Society of Anesthesiologists (ASA) physical status classification of I to III who are scheduled to undergo surgical treatment for breast cancer. Patients with preoperative depressive symptoms, defined as a HAMD-17 score ≥7, will be eligible for inclusion. This threshold was selected to identify participants with clinically relevant depressive symptoms at baseline rather than to indicate a diagnosis of major depressive disorder or a specific severity category. All participants should demonstrate adequate cognitive capacity to understand the study procedures and assessment instruments, as well as the ability to communicate effectively with research staff. Written informed consent will be obtained from all participants prior to enrollment, and only those who express a clear willingness to participate will be included in the study.

### 2.4 Exclusion criteria

Participants will be excluded if they refuse or are unable to provide written informed consent, or if they have an ASA physical status classification greater than III. Additional exclusion criteria will include a history of diagnosed neuropsychiatric disorders or neurological diseases that may affect study assessments, as well as chronic use of psychotropic medications, including but not limited to corticosteroids, antidepressants, and anxiolytics. Individuals with a history of epilepsy or active seizure episodes will also be excluded. Furthermore, patients with cranial or scalp injuries, severe cardiovascular or cerebrovascular diseases, or local infection at the intended stimulation site will be deemed ineligible. The presence of metallic implants in the body, such as cardiac pacemakers, intraocular metallic implants, a history of intracranial metal implantation surgery, cochlear implants, or other similar devices, will also constitute grounds for exclusion.

### 2.5 Dropout criteria

Participants may withdraw from the study at any time without providing a reason and without any impact on their standard clinical care.

Participants will be considered withdrawn from the study under the following conditions: (1) voluntary withdrawal of consent; (2) significant protocol deviation affecting intervention adherence or outcome assessment; (3) initiation of concomitant treatments that are likely to interfere with study outcomes, as judged by the investigators; or (4) occurrence of severe intraoperative or postoperative complications that preclude continuation of study procedures, including hemodynamic instability, major hemorrhage, anaphylaxis, cardiac arrest, or other life-threatening events.

Participants who miss postoperative assessments and cannot be contacted within 7 days after surgery will be classified as lost to follow-up. A single additional contact attempt will be made within 48 hours before final classification.

All withdrawal and discontinuation reasons will be documented. Participants who withdraw or are lost to follow-up will be included in the intention-to-treat analysis whenever outcome data are available, and missing data will be handled using appropriate statistical methods as specified in the statistical analysis plan.

### 2.6 Randomization and blinding

Participants will be randomly assigned to either the active rTMS group or the sham-rTMS group using a computer-generated random sequence with block randomization (block size = 4). The random sequence will be generated by an independent researcher who is not involved in participant recruitment, intervention administration, or outcome assessment.

Allocation concealment will be maintained using sequentially numbered, opaque, sealed envelopes prepared according to the randomization sequence. These envelopes will be stored by an independent researcher and opened only immediately before the intervention after eligibility confirmation and completion of baseline assessments. The allocation information will not be accessible to investigators responsible for recruitment, outcome assessment, or data analysis until the prespecified stage of the trial.

Participants, outcome assessors, and data analysts will be blinded to group allocation. Participants will be blinded to treatment allocation through standardized active and sham procedures that are matched in appearance, duration, and operation process. The rTMS operators responsible for delivering stimulation cannot be blinded due to the nature of the intervention; however, they will not participate in outcome assessment or statistical analysis. Independent outcome assessors who are unaware of allocation information will conduct all clinical evaluations. Outcome assessors will receive standardized training before study initiation to ensure consistency in assessment procedures. Statistical analyses will be performed using coded treatment groups, and the allocation code will remain concealed until completion of the primary outcome analysis.

To assess the effectiveness of participant blinding, a brief treatment-allocation assessment will be conducted immediately after completion of the second intervention session on the day of surgery. Participants will be asked to indicate which treatment they believe they received: active rTMS, sham rTMS, or uncertain. This assessment will be used to evaluate the credibility of the sham procedure and the potential influence of treatment expectancy on the interpretation of clinical outcomes. The results of the participant blinding assessment will be reported alongside the primary outcome.

### 2.7 Unblinding

Unblinding will only be permitted when knowledge of treatment allocation is essential for the clinical management of a participant, such as in the case of serious adverse events potentially related to the intervention, or when required for safety reporting. Requests for unblinding will be reviewed and documented by the principal investigator or designated safety monitor. The reason, timing, and personnel involved in each unblinding event will be recorded. Routine outcome assessment and data analysis will remain blinded whenever possible.

### 2.8 Anesthesia

All participants will undergo standardized general anesthesia according to institutional protocols. Anesthetic management, including induction, maintenance, intraoperative monitoring, and postoperative recovery, will be performed by experienced anesthesiologists.

To minimize potential confounding effects on postoperative depressive symptoms and neurobiological outcomes, relevant perioperative anesthesia-related variables will be recorded, including type and duration of anesthesia, intraoperative medication use, duration of surgery, and intraoperative complications. Postoperative analgesic management will also be standardized according to institutional protocols, and relevant analgesic information will be documented for adjustment in subsequent analyses when appropriate.

### 2.9 Study interventions

For patients scheduled for breast cancer surgery at our institution, eligible participants will be screened the day before surgery according to the predefined inclusion criteria, including a preoperative HAMD-17 score ≥7, indicating clinically relevant depressive symptoms at baseline.

Participants will then be randomly assigned to receive either active rTMS or sham rTMS targeting the left dorsolateral prefrontal cortex (DLPFC). Participants will receive two sessions of rTMS during the perioperative period. The first session will be administered one day before surgery, and the second session will be delivered before surgery on the day of operation, according to the standardized protocol described in **Table 1**. The intervention protocol was designed according to established safety guidelines for clinical rTMS application.

**Table 1:** Schedule of enrollment, interventions, and assessments.

Study period	Screening	Baseline (preoperative assessment)	Preoperative day 1	Day of surgery	Postoperative day 1	Postoperative day 3	Postoperative day 7	3-month follow-up
Time point	Before enrollment	T0	T1	T2	T3	T4	T5	T6
Enrollment								
Eligibility screening	×							
Informed consent	×							
Demographic characteristics and medical history		×						
Cancer-related clinical characteristics		×						
Baseline depressive symptoms (HAMD-17)		×						
Randomization		×						
Interventions								
Active rTMS intervention			×	×				
Sham rTMS intervention			×	×				
Outcome assessments								
Primary outcome: incidence of postoperative depression (PD) (HAMD-17 ≥7)		×			×	×	×	×
Participant blinding assessment				×				
Continuous depressive symptom severity (HAMD-17 score)		×			×	×	×	×
Pain assessment (NRS)					×	×		
Postoperative adverse events				×	×	×	×	×
Frailty assessment (FRAIL scale)					×	×	×	×
Sleep quality assessment (PSQI)					×	×	×	×
Serum BDNF measurement		×			×			

Pre, Preoperative day; POD, Postoperative day; HAMD, Hamilton Depression Rating Scale; FRAIL, Fatigue, Resistance, Ambulation, Illness, and Loss of weight; PSQI, Pittsburgh Sleep Quality Index; NRS, Numerical Rating Scale; BDNF, Brain-derived neurotrophic factor.

The investigators responsible for delivering the intervention will not participate in outcome assessment or data analysis to minimize potential bias. Continuous monitoring will be performed during stimulation. If a participant reports intolerable discomfort or adverse effects, the stimulation will be immediately discontinued, and appropriate clinical evaluation will be conducted.

#### 2.9.1 Active-rTMS

Repetitive transcranial magnetic stimulation (rTMS) will be delivered using a CCY-1 magnetic stimulator (or equivalent clinically approved device) equipped with a figure-of-eight coil. Stimulation will target the left dorsolateral prefrontal cortex (DLPFC), which is localized using the Beam F3 method to ensure reproducible and anatomically standardized positioning across participants.

Resting motor threshold (RMT) will be determined for each participant by identifying the minimum stimulus intensity required to produce visible abduction of the contralateral abductor pollicis brevis muscle in at least 5 out of 10 consecutive stimulations.

Active rTMS will be administered using high-frequency stimulation at 10 Hz with an intensity of 120% of the individual RMT. Each session will consist of 40 trains, with each train delivering 50 pulses (total 2000 pulses per session). Trains will be separated by 25-second inter-train intervals, resulting in an approximate total session duration of 20–25 minutes.

Participants will receive two rTMS sessions in total: one session on the day before surgery and one session before surgery on the day of operation.

#### 2.9.2 Sham-rTMS

For the sham condition, the coil will be positioned at the same scalp location using an identical device setup. Sham stimulation will reproduce acoustic noise and scalp sensations using a tilted coil positioning (45° angle) with a built-in sham mode where applicable, without delivering a physiologically effective magnetic field to the cortex. This approach is designed to maintain participant blinding while minimizing cortical stimulation.

Participants will remain seated comfortably during stimulation and will be continuously monitored for adverse events, including headache, scalp discomfort, dizziness, or other neurological symptoms. Any adverse events will be recorded and evaluated according to predefined safety monitoring procedures.

### 2.10 Outcomes

#### 2.10.1 Primary outcome

The primary outcome is the incidence of postoperative depression (PD) at postoperative day 3, consistent with the registered protocol. Operationally, this endpoint is defined as the proportion of participants with a postoperative day 3 HAMD-17 score ≥7. Because all participants enrolled in this trial are required to have preoperative depressive symptoms (HAMD-17 ≥7), this endpoint does not represent the occurrence of depression among previously asymptomatic individuals. Instead, it represents the proportion of participants who meet the predefined HAMD-17 threshold for postoperative depression at the prespecified postoperative assessment time point.

HAMD-17 will be used to assess depressive symptom severity at each time point. The scale is a clinician-rated instrument widely used in perioperative and psychiatric research. For descriptive purposes and secondary analyses, depressive symptom severity will be categorized according to HAMD-17 scores as follows: 0–6 indicates no clinically relevant depressive symptoms, 7–16 indicates mild depressive symptoms, 17–23 indicates moderate depressive symptoms, and ≥24 indicates severe depressive symptoms.

#### 2.10.2 Secondary outcomes

Secondary outcomes will be assessed at prespecified time points, including baseline before surgery, postoperative day 1, postoperative day 3, postoperative day 7, and the 3-month follow-up visit. The detailed assessment schedule is presented in **Table 1**.

Longitudinal changes in depressive symptom severity will be evaluated using continuous HAMD-17 scores at the same time points. The HAMD-17 is a clinician-administered scale widely used to quantify depressive symptom severity. Total scores range from 0 to 52, with higher scores indicating greater severity.

Frailty status will be assessed using the FRAIL scale, which evaluates five domains: fatigue, resistance, ambulation, illness, and weight loss. Each domain is scored as 0 or 1, yielding a total score ranging from 0 to 5. A score of 0 indicates robust status, 1–2 indicates pre-frailty, and 3–5 indicates frailty.

Sleep quality will be measured using the Pittsburgh Sleep Quality Index (PSQI) at postoperative day 1, 3, 7, and 3 months. The PSQI consists of 19 self-rated items and 5 clinician-rated items, generating seven component scores and a global score ranging from 0 to 21. Higher scores indicate poorer sleep quality, with a total score >5 generally indicating significant sleep disturbance.

Postoperative pain intensity will be assessed using the 11-point Numerical Rating Scale (NRS) within 72 hours after surgery, where 0 indicates no pain and 10 represents the worst imaginable pain. Pain severity will be categorized as mild (1–3), moderate (4–6), and severe (7–10).

Serum BDNF levels will be measured at baseline before intervention and postoperative day 1 in a randomly selected subset of 30 participants from each group. Participants included in this biomarker substudy will be randomly selected from the enrolled participants after completion of baseline assessment. This BDNF analysis represents an exploratory biomarkers analysis within the main trial protocol. Venous blood samples will be collected, centrifuged promptly, and stored at −80 °C until analysis. Serum BDNF concentrations will be quantified using enzyme-linked immunosorbent assay (ELISA) according to manufacturer instructions.

### 2.11 Adverse events and safety

Adverse events (AEs) related to rTMS, including headache, dizziness, transient vertigo, scalp discomfort, and tinnitus, will be systematically monitored and recorded throughout the study period. Serious adverse events (SAEs) and unexpected medical occurrences will be documented in accordance with Good Clinical Practice (GCP) guidelines and reported to the institutional ethics committee and relevant regulatory authorities in a timely manner.

An independent data monitoring process will be implemented to ensure participant safety and data integrity. All safety-related assessments will be conducted in a standardized manner without suggestive or leading questioning. In the event of any intolerable adverse reaction, rTMS stimulation will be immediately discontinued, and appropriate clinical evaluation and management will be provided. The safety profile of rTMS in perioperative populations will be continuously evaluated.

### 2.12 Data collection and management

Trial data will be collected using standardized case report forms (CRFs). Preoperative data will include demographic characteristics, current health status, medical history, type of surgery, income level, family cancer history, and HAMD-17 scores. Intraoperative data will include blood loss, urine output, and the duration of surgery and anesthesia. Postoperative data will include depressive symptom severity assessed using the HAMD-17, pain intensity assessed using the numerical rating scale (NRS), sleep quality assessed using the Pittsburgh Sleep Quality Index (PSQI), and other prespecified clinical variables. Questionnaire and clinical data will initially be recorded on paper CRFs and subsequently entered into a secure electronic database by trained research personnel. Data entry will follow standardized procedures, and data accuracy and completeness will be verified through regular quality checks. Access to the electronic database will be restricted to authorized study personnel, and data will be securely stored to maintain confidentiality and integrity. Access to data entry, processing, and analysis will be restricted to authorized personnel according to their assigned study responsibilities. For biomarker analyses, collected blood samples will be processed according to the prespecified laboratory procedures and stored under the specified conditions until analysis.

### 2.13 Sample size

The sample size calculation was based on preliminary clinical data from our institution. In these preliminary data, the proportion of participants meeting the predefined HAMD-17 threshold (≥7) for postoperative depression (PD) at postoperative day 3 was approximately 62.54%.

Assuming that perioperative rTMS would achieve a 35% relative reduction in the incidence of postoperative depression (PD), the expected incidence was estimated to decrease from 62.54% in the control group to 40.65% in the intervention group. This corresponds to an absolute risk reduction of 21.89 percentage points.

Using a two-sided significance level of 0.05 and 80% power, the required sample size was calculated based on comparison of these two proportions. Considering a potential dropout rate of 10%, the final sample size was determined to be 182 participants.

### 2.14 Statistical analysis

All statistical analyses will be conducted using a two-sided significance level of 0.05. Continuous variables will be summarized as mean ± standard deviation or median with interquartile range, depending on data distribution, while categorical variables will be presented as frequencies and percentages. Normality will be assessed using the Shapiro-Wilk test.

The primary analysis will follow the intention-to-treat (ITT) principle. Missing data will be handled using multiple imputation under the assumption of missing at random (MAR). A sensitivity analysis will be performed using complete-case data to assess robustness of the findings.

The primary outcome, defined as the proportion of participants meeting the predefined HAMD-17 threshold (≥7) for postoperative depression (PD) at postoperative day 3, will be compared between groups using chi-square tests or Fisher’s exact tests, as appropriate. Effect sizes will be reported as odds ratios (ORs) with 95% confidence intervals (CIs). The binary definition of postoperative depression (HAMD-17 ≥7) will be used exclusively for the prespecified primary endpoint analysis.

As a secondary exploratory analysis, continuous HAMD-17 scores measured at baseline and postoperative day 1, day 3, day 7, and 3 months will be analyzed using a linear mixed-effects model (LMM) to characterize longitudinal changes in depressive symptom severity. The model will include fixed effects for group (active rTMS vs sham rTMS), time, and group × time interaction. A random intercept for each participant will be included to account for within-subject correlations across repeated measurements. An unstructured covariance matrix will be used if model convergence allows; otherwise, alternative covariance structures (e.g., compound symmetry) will be considered based on Akaike information criterion (AIC) comparison.

The sample size calculation was based on the primary binary endpoint and was not designed to provide specific statistical power for the longitudinal mixed-effects model analysis. Therefore, the LMM results will be interpreted as exploratory evidence regarding treatment-related changes in depressive symptom trajectories rather than confirmatory evidence. Model estimates will be reported as least squares means with 95% confidence intervals. *Post hoc* pairwise comparisons will be adjusted using Bonferroni correction.

To reduce potential confounding, prespecified covariates will be included in adjusted analyses based on clinical relevance and prior literature. These covariates include age, body mass index (BMI), baseline HAMD-17 score, type of surgical procedure, and perioperative anesthesia duration. For the primary binary outcome, adjusted logistic regression models will be used to estimate adjusted odds ratios (aORs) and 95% confidence intervals. For longitudinal outcomes, covariates will be incorporated into the mixed-effects models as fixed effects. No *post hoc* covariate selection will be performed to avoid data-driven bias.

For serum BDNF analysis, Pearson or Spearman correlation analysis will be performed to explore the association between changes in BDNF levels and changes in depressive symptom. As this biomarker analysis is exploratory, no separate sample size calculation will be performed, and findings will be interpreted as hypothesis-generating. Subgroup analyses may be conducted based on baseline depressive symptom severity; however, these analyses will also be considered exploratory and hypothesis-generating.

## 3 Discussion

Surgical treatment remains the cornerstone of management for breast cancer (20). However, the perioperative period represents a vulnerable phase for patients with breast cancer, during which surgical stress and psychological burden may contribute to depressive symptoms and impaired recovery (21), thereby adversely affecting recovery trajectories, quality of life (QoL), and treatment adherence.

To evaluate the efficacy and safety of repetitive transcranial magnetic stimulation (rTMS) in reducing the incidence of postoperative depression (PD) and improving depressive symptom severity in patients with breast cancer, this prospective, randomized, sham-controlled trial was designed. Depressive symptoms following mastectomy or breast-conserving surgery represent a clinically relevant but often under-recognized perioperative complication (22). Although pharmacological and psychotherapeutic interventions are available, their use in perioperative oncology populations may be limited by delayed onset of effect, adverse effects, or logistical constraints (23). In this context, rTMS offers a non-invasive neuromodulation approach that may modulate dysfunctional cortical and subcortical networks implicated in affective regulation (24).

We hypothesize that active rTMS will reduce the incidence of postoperative depression (PD) at postoperative day 3 compared with sham stimulation. In addition, active rTMS is expected to improve longitudinal depressive symptom severity assessed by continuous HAMD-17 scores and secondary clinical outcomes. The potential neurobiological mechanisms may involve modulation of prefrontal-limbic circuitry and downstream effects on neuroplasticity-related pathways; however, these mechanisms remain exploratory and require confirmation in future multimodal neuroimaging studies (25).

The use of a standardized high-frequency rTMS protocol targeting the left dorsolateral prefrontal cortex (lDLPFC) is consistent with established treatment paradigms for depressive disorders, thereby enhancing methodological reproducibility (26). By focusing on a perioperative breast cancer population and incorporating multidimensional outcomes including postoperative depression incidence, depressive symptom severity, frailty, sleep quality, pain, and quality of life (27), this study extends current evidence toward a more comprehensive understanding of neuromodulation in perioperative affective symptom management.

Finally, several limitations should be acknowledged. First, the study is conducted at a single institution, which may limit the generalizability of the findings to other populations and clinical settings. Second, a fixed stimulation protocol is used to ensure intervention consistency, which may not fully account for individual differences in cortical excitability and may limit the generalizability of the stimulation parameters (28). Third, although the sham-rTMS procedure is designed to maintain participant blinding, the use of a 45° tilted-coil technique may allow some participants to infer their treatment allocation, potentially introducing expectancy-related bias. To address this concern, participant treatment-allocation beliefs will be assessed at the end of the intervention period. Finally, variability in postoperative analgesic use may influence patient-reported symptoms and HAMD-17 assessments. Although analgesic use will be documented and considered as a covariate in adjusted analyses, residual confounding cannot be completely excluded. More broadly, other unmeasured perioperative factors may also contribute to residual confounding despite efforts to standardize the surgical and anesthetic protocols.

Although rTMS has demonstrated antidepressant effects in major depressive disorder, evidence regarding its role in perioperative depressive symptom management in breast cancer populations remains limited. This study aims to determine whether perioperative rTMS can reduce the incidence of postoperative depression (PD) and improve depressive symptom severity in this clinical context. The findings may provide preliminary evidence to inform future neuromodulation strategies for perioperative affective symptom management in oncological settings.
