# Supplementary material for: Efficacy of perioperative repetitive transcranial magnetic stimulation for postoperative depression in breast cancer patients: study protocol for a single-center randomized controlled trial
Source: Front Psychiatry. 2026 Sep 14;17:1923066. doi: 10.3389/fpsyt.2026.1923066 (PMC13617041; doi:10.3389/fpsyt.2026.1923066)
Supplement: Supplementary file 1 [file Supplementaryfile1.pdf]

SPIRIT 2013 Checklist: Recommended items to address in a clinical trial protocol

Completed checklist for: Efficacy of repetitive transcranial magnetic stimulation for perioperative depressive symptoms in breast cancer patients: study protocol for a single-center randomized controlled trial

Item	SPIRIT item	Where addressed	Description
1	Title	Title page	Descriptive title identifies population (breast cancer patients), intervention (rTMS), and study design (randomized controlled trial).
2a	Trial registration	Section 2.1 Study design; Abstract	Registered at China Clinical Trial Registration Center (ChiCTR2500097808).
2b	WHO Trial Registration Data Set	Trial registration record and protocol	Trial registration information provided according to registry requirements.
3	Protocol version	Manuscript title page / protocol information	Current protocol version is provided in the submitted manuscript version.
4	Funding	Declarations/Funding statement	Funding source and support information are reported.
5a	Protocol contributors	Author information	Names, affiliations, and roles of contributors are provided.
5b	Trial sponsor	Declarations	Sponsor information is provided where applicable.
5c	Sponsor/funder role	Declarations	Roles of sponsor/funders in design, conduct, analysis, and publication are described.
5d	Trial oversight	Methods sections	Responsibilities for investigators, intervention delivery, assessment, and data management are described.
6a	Background and rationale	Introduction	Scientific background,

			clinical rationale, and evidence gap are described.
6b	Choice of comparator	Introduction; Methods	Rationale for sham-rTMS comparator is described.
7	Objectives	Introduction; Abstract	Primary and secondary objectives and hypothesis are specified.
8	Trial design	Section 2.1 Study design	Prospective, single-center, randomized, sham-controlled parallel-group design is described.
9	Study setting	Section 2.1 Study design	Trial setting and study site are described.
10	Eligibility criteria	Section 2.2 Participants and eligibility criteria	Inclusion and exclusion criteria are provided.
11a	Interventions	Section 2.3 Intervention	Active and sham rTMS procedures, timing, parameters, and delivery are described.
11b	Intervention modification/discontinuation	Sections 2.5 and 2.11	Withdrawal criteria and adverse event management are described.
11c	Adherence strategies	Section 2.3 Intervention	Intervention delivery procedures are standardized.
11d	Concomitant care	Sections 2.8 and statistical analysis	Perioperative care and relevant covariates are documented.
12	Outcomes	Section 2.10 Outcomes	Primary and secondary outcomes, measurements, and assessment time points are specified.
13	Participant timeline	Table 1; Figure 1	Schedule of enrollment, intervention, and assessments is presented.
14	Sample size	Section 2.13 Sample size calculation	Sample size calculation and assumptions are

			described.
15	Recruitment	Section 2.14 Recruitment	Recruitment strategy is described.
16a	Allocation sequence generation	Section 2.6 Randomization and blinding	Computer-generated random sequence with block randomization is described.
16b	Allocation concealment	Section 2.6 Randomization and blinding	Sequentially numbered opaque sealed envelopes are described.
16c	Implementation	Section 2.6 Randomization and blinding	Independent researcher generates sequence and maintains concealment.
17a	Blinding	Section 2.6 Randomization and blinding	Participant, assessor, and analyst blinding procedures are described.
17b	Similarity of interventions	Section 2.3 Intervention	Sham stimulation procedures are described.
18a	Data collection methods	Sections 2.10 and 2.12	Outcome assessment procedures and data collection methods are described.
18b	Participant retention	Section 2.5 Dropout criteria	Withdrawal and follow-up procedures are described.
19	Data management	Section 2.12 Data management	Data entry, storage, confidentiality, and quality assurance procedures are described.
20a	Statistical methods	Section 2.14 Statistical analysis	Analysis of primary and secondary outcomes is described.
20b	Additional analyses	Section 2.14 Statistical analysis	Exploratory subgroup and biomarker analyses are described.
20c	Analysis population and missing data	Section 2.14 Statistical analysis	Intention-to-treat analysis and missing data handling are described.
21a	Data monitoring committee	Section 2.11 Adverse	Need for independent

		events	DMC is addressed according to trial risk.
21b	Interim analyses	Section 2.14 Statistical analysis	No interim efficacy analysis is planned.
22	Harms	Section 2.11 Adverse events	Adverse event monitoring and management are described.
23	Auditing	Ethics and monitoring sections	Trial conduct monitoring procedures are described.
24	Research ethics approval	Ethics statement	Ethics approval information is provided.
25	Protocol amendments	Ethics and dissemination	Procedures for communicating protocol modifications are described.
26a	Consent	Ethics statement / informed consent	Written informed consent procedures are described.
26b	Additional consent for specimens	BDNF substudy section	Consent for biological sample collection is described.
27	Confidentiality	Data management section	Participant confidentiality protection is described.
28	Declaration of interests	Declarations	Competing interests are reported.
29	Access to data	Data availability/declarations	Data access procedures are described.
30	Ancillary/post-trial care	Ethics statement	Post-trial care considerations are described.
31a	Dissemination policy	Declarations	Publication and dissemination plans are described.
31b	Authorship	Declarations	Authorship follows journal and research standards.
31c	Public access to data/protocol	Data availability statement	Data sharing plans are described.
32	Informed consent materials	Supplementary materials	Consent documentation is available.
33	Biological specimens	BDNF substudy section;	Serum BDNF samples

		Table 1	are collected at baseline and postoperative day 1, processed, stored, and analyzed as exploratory biomarker outcomes.
